# Treatment-Related Death in Patients with Small-Cell Lung Cancer in Phase III Trials over the Last Two Decades

**DOI:** 10.1371/journal.pone.0042798

**Published:** 2012-08-06

**Authors:** Nobuaki Ochi, Katsuyuki Hotta, Nagio Takigawa, Isao Oze, Yoshiro Fujiwara, Eiki Ichihara, Akiko Hisamoto, Masahiro Tabata, Mitsune Tanimoto, Katsuyuki Kiura

**Affiliations:** 1 Department of Respiratory Medicine, Okayama University Hospital, Okayama, Japan; 2 Department of General Internal Medicine 4, Kawasaki Hospital, Kawasaki Medical School, Okayama, Japan; National Taiwan University Hospital, Taiwan

## Abstract

**Introduction:**

Treatment-related death (TRD) remains a serious problem in small-cell lung cancer (SCLC), despite recent improvements in supportive care. However, few studies have formally assessed time trends in the proportion of TRD over the past two decades. The aim of this study was to determine the frequency and pattern of TRD over time.

**Methods:**

We examined phase 3 trials conducted between 1990 and 2010 to address the role of systemic treatment for SCLC. The time trend was assessed using linear regression analysis.

**Results:**

In total, 97 trials including nearly 25,000 enrolled patients were analyzed. The overall TRD proportion was 2.95%. Regarding the time trend, while it was not statistically significant, it tended to decrease, with a 0.138% decrease per year and 2.76% decrease per two decades. The most common cause of death was febrile neutropenia without any significant time trend in its incidence over the years examined (p = 0.139). However, deaths due to febrile neutropenia as well as all causes in patients treated with non-platinum chemotherapy increased significantly (p = 0.033).

**Conclusions:**

The overall TRD rate has been low, but not negligible, in phase III trials for SCLC over the past two decades.

## Introduction

Chemotherapy is the mainstay of treatment for small-cell lung cancer (SCLC); it is widely accepted that patients with limited-stage SCLC (LD-SCLC) have prolonged survival with systemic chemotherapy when combined with thoracic irradiation [1], [2]. Even in patients with extended-stage SCLC (ED-SCLC), chemotherapy has yielded a survival advantage, with a median survival time of over 1 year [3]–[5].

However, chemotherapy-related toxicity sometimes leads to treatment-related death (TRD) and often to deterioration in the patient's quality-of-life. Thus, toxicity profile information as well as data on efficacy from phase III trials are essential for a full discussion by physicians and patients in clinical practice.

Although there have been many phase III trials involving SCLC patients investigating the efficacy of chemotherapy, few studies have focused specifically on the frequency or pattern of chemotherapy-related fatal toxicity. The aim of this study was to clarify this issue and its time trends over the last two decades, using data from phase III systemic treatment trials that included about 25,000 patients.

## Materials and Methods

### Trials

We conducted a search for trials reported from January 1990 to March 2010. To avoid publication bias, we identified both published and unpublished trials through a computer-based search of the PubMed database and abstracts from ten past conferences of the American Society of Clinical Oncology, European Society for Medical Oncology, and the International Association for the Study of Lung Cancer. We used the following search terms: *lung cancer, chemotherapy, and randomized controlled study*. The search was extended by a thorough examination of reference lists from original articles, review articles, relevant books, and the Physician Data Query registry of clinical trials.

### Trial Selection

Phase III trials that investigated the systemic treatment of previously untreated LD- and ED-SCLC patients with cytotoxic agents were eligible. Trials designed with concurrent thoracic radiotherapy (TRT) or prophylactic cranial irradiation sequentially after the induction of chemotherapy were included. Some phase III trials incorporated patients with both LD- and ED-SCLC. Trials that provided data for TRD in each report were included. Clinical trials of salvage chemotherapy (second-line or later-setting) were ineligible.

### Data collection and data items

To avoid bias in the data abstraction process, four medical oncologists (NO, IO, YF, and KH), three of whom (NO, IO, and KH) hold board certificates in medical oncology, abstracted data independently from the trials and subsequently compared the results, as described previously [5]–[13].

The following information was obtained from each report: year of trial initiation, year of publication, number of patients enrolled and randomized, proportion of patients with a good performance status (PS), proportion of male patients, median age of patients, number of chemotherapeutic regimens, description of the administration of concurrent or sequential thoracic irradiation, treatment regimens in each treatment arm, total number of patients with TRD, cause of TRD in each treatment arm, and the definition of LD or ED (the definitions of LD- and ED-SCLC varied somewhat from trial to trial, but we did not reallocate each patient strictly in this study because we were unable to access individual patient data).

All data were checked for internal consistency, and disagreements were resolved by discussion among the investigators.

### Definition of TRD

We defined TRD should satisfy all the followings:

1. death occurring within 4 weeks of the completion of treatment,
2. death ‘possibly,’ ‘probably,’ or ‘definitely’ related to treatment reported by investigators, as defined previously [6], [7].
3. death without clear evidence of any other cause of death (i.e., disease progression)

We also defined febrile neutropenia (FN)-associated death, the most common cause of fatal toxicity during chemotherapy [7], as death related to fever of unknown origin without clinically or microbiologically documented infection with absolute neutrophil count <1.0×10^9^/L and fever >38.3°C. In general, more recent trials included in this study defined TRD and/or FN-related death clearly in their reports. However, previous studies tended to be left their definitions vague and not to state them specifically. In response to that situation, we tried best to contact the principal authors of the reports for each trial to clarify this and to get precise number of TRD and FN-related death. In case we could not obtain any additional information despite these intensive efforts, we accepted the number of TRDs and FN-related deaths as was described in those reports.

The data we collected from each trial also included the number stratified by representative cause of toxic death other than FN-related one. On the basis of our previous study, the causes of TRD were collected as follows [7]: FN, hemorrhage, renal failure, central nervous system (CNS) disorder, cardiovascular disorder and pulmonary disorder. Hemoptysis, upper and lower gastrointestinal hemorrhages, and disseminated intravascular coagulation-related hemorrhage were all categorized as “hemorrhage”, while both CNS ischemia and hemorrhage were classified as “CNS disorder”. “Cardiovascular disease” included ischemia, infarction, or embolism in any organ other than the CNS (i.e., myocardial infarction and pulmonary embolism). “Pulmonary disorders” included all pulmonary diseases other than pulmonary embolisms, including infection without neutropenia (i.e., pneumonia) [7].

### Quantitative Data Synthesis

The incidence of TRD was defined as the number of TRDs divided by the number of randomized patients. To derive the annual change in TRD incidence during the observation period, we calculated this number for each year of publication. The association between the year of publication and incidence of TRD was analyzed using linear regression analysis, weighted by sample size. All *p*-values corresponded to two-sided tests, and significance was set at p<0.05. Statistical analyses were conducted using STATA software (ver. 10; StataCorp, College Station, TX, USA).

## Results

### Trial flow and characteristics of the eligible trials

**Figure 1:**
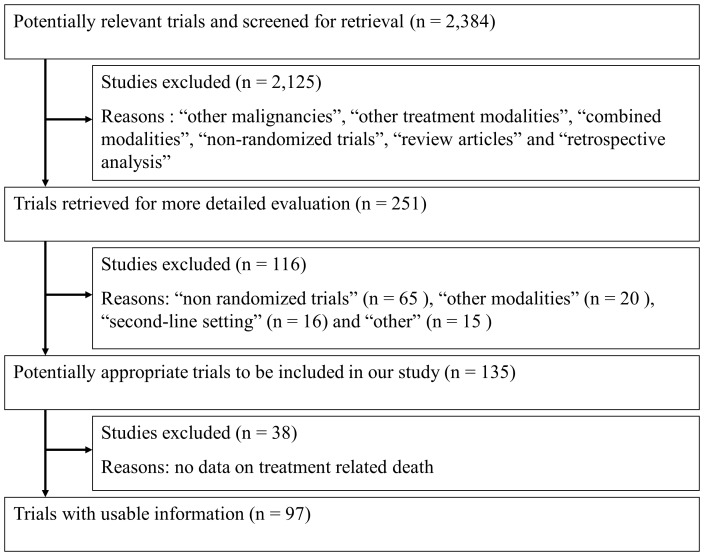
PRISMA flow diagram showing the progress of trials through the review.

Figure 1 shows a flow chart for this study. In total, we identified 97 trials as a result of computer-based and manual searches (File S1). In total, 24,152 patients were randomized and allocated to 208 treatment arms. Table 1 shows the characteristics of all eligible trials. The median proportion of randomized patients with a good PS (0 or 1) and that of male patients in all trials was 80.0 and 71.0%, respectively. Most trials had two chemotherapy arms (86.6%). The number of trials designed to assign TRT in addition to chemotherapy was 53 (54.6%).

**Table 1:** Characteristics of the 97 trials.

Variables	Values
Proportion of randomized patients with a good performance status* (%)	
<80	43.3
80–90	27.8
>90	19.6
Median proportion (range)	80.0 (23.0–100)
Proportion of male patients (%)	
<80	63.0
80–90	19.0
>90	12.0
Median proportion (range)	71.0 (41.0–99.0)
Type of disease stage included (LD only/others)	19/78
No. of treatment arms	
2	84
3	10
4	3
Published year (median; range)	1997 (1990–2009)
Trials designed to assign TRT (yes/no)	53/44

* A good performance status (PS) was defined as an Eastern Cooperative Oncology Group (ECOG) PS of 0 or 1.

LD, limited disease; TRT, thoracic radiotherapy.

**Figure 2:**
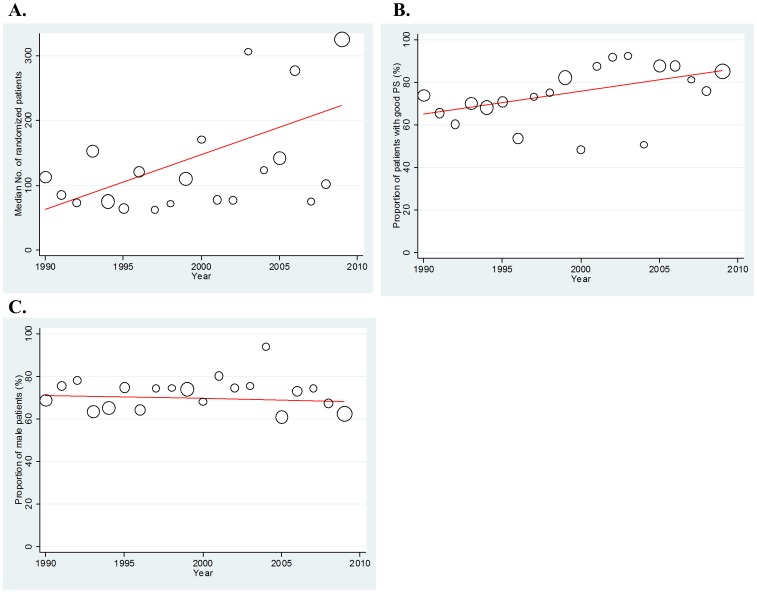
Time trends in the demographics of patients randomized in phase III trials. A good PS was defined as an Eastern Cooperative Oncology Group (ECOG) PS of 0 or 1. All analyses were weighted by sample size. A. Median number of randomized patients. B. Proportion of patients with a good PS. C. Proportion of male patients.

The median number of randomized patients and proportion of patients with a good PS in each trial increased significantly, with 8.489 patients and 1.075% per year, respectively (regression coefficients = 8.489 and 1.075, corresponding to an 8.489 and 1.075% increase per year; p = 0.003 and 0.009, respectively; Fig. 2A and B). The proportion of male patients, however, showed no particular change over time (Fig. 2C).

### Time trends in treatment regimens

**Figure 3:**
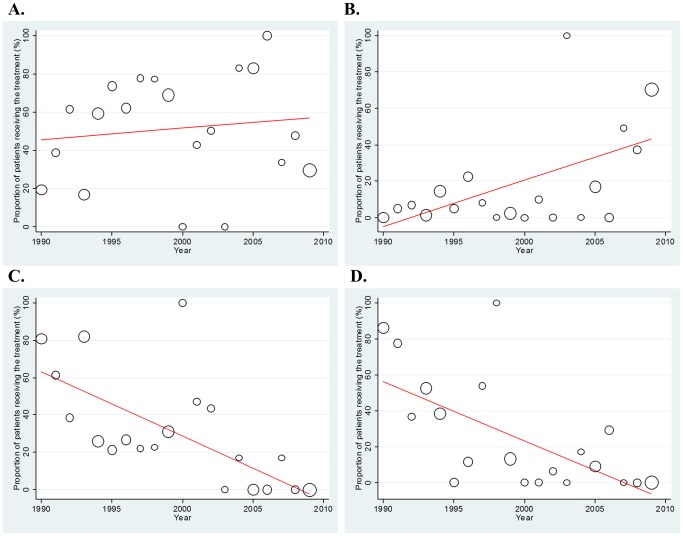
Time trends in chemotherapeutic regimen. All analyses were weighted by sample size. A. Cisplatin-containing regimen. B. Carboplatin-containing regimen. C. Non-platinum regimen. D. CAV (cyclophosphamide, doxorubicin and vincristine)-based regimen.

Figure 3 shows the changes in treatment regimens over the past two decades. Regarding platinum-based regimens, the proportion of cisplatin use was largely constant during the period (regression coefficient = 0.599, corresponding to a 0.599% increase per year; p = 0.549; Fig. 3A), while carboplatin (CBDCA)-containing regimens increased yearly (regression coefficient = 2.527 [2.527% increase per year]; p = 0.004; Fig. 3B). In contrast, the use of non-platinum combination regimens and that of cyclophosphamide, doxorubicin, and vincristine (CAV)-based regimens decreased significantly during the two decades, at 3.438% (p<0.001) and 3.300% (p = 0.001) per year, respectively (Fig. 3C and D).

### Time trends in overall TRD incidence

Data for the calculation of the overall incidence of TRD were available for all 97 trials with their 208 chemotherapy arms (24,152 patients), whereas information about the causes of death were provided for 154 arms (74.0%; 17,570 patients). The crude TRD proportion in the overall cohort was 2.95%. Of these, the most common cause of death was febrile neutropenia (FN) (1.25%), followed by pulmonary disorder (0.45%). The crude TRD proportions of other causes collected in this study were very low compared with FN and pulmonary disorder (hemorrhage 0.03%, renal failure 0.05%, CNS disorder 0.02%, cardiovascular disorder 0.12%, and others 0.18%).

**Figure 4:**
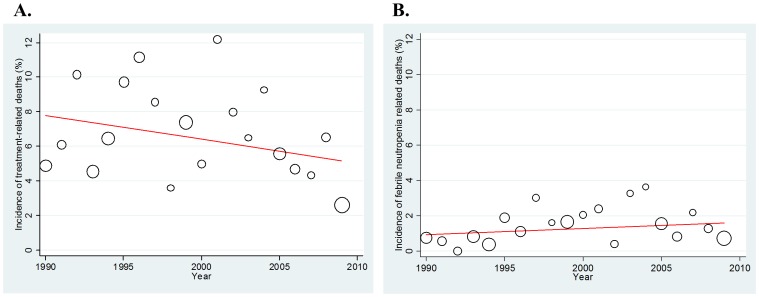
Time trend in the incidence of TRDs (treatment-related deaths). The analysis was weighted by sample size. A. Overall incidence of TRDs. B. Incidence of FN (febrile neutropenia)-related TRDs.

Next, we assessed the time trends in TRD incidence. It was stable over the last two decades, with no statistically significant difference (regression coefficient = −0.138; p = 0.15). This corresponds to a 0.138% decrease per year; however, it does mean that, theoretically, the TRD incidence decreased by 2.76% per two decades (Fig. 4A). Further, we assessed which clinical factor affected this time trend (Table 2). In most clinical settings, there was no particular difference in the time trend, whereas, interestingly, when limited to patient cohorts treated with a non-platinum regimen, there was a significant increase in TRD incidence (0.146% increase per year; p = 0.033). We observed no significant increase or decrease in TRD incidence with other treatment regimens, including cisplatin-, carboplatin-, and CAV-based regimens (p = 0.270, 0.390, and 0.570, respectively).

**Table 2:** Time trends in the incidence of treatment-related deaths in various clinical settings (simple regression analysis).

Subgroups	Regression coefficient	p-value
Trials designed to assign TRT		
Yes	−0.021	0.820
No	−0.290	0.073
Type of disease stage included		
LD only	−0.012	0.873
Other	0.049	0.394
Proportion of randomized patients with a good performance status (%)**		
≥80	0.018	0.681
<80	−0.774	0.233
Proportion of male patients (%)**		
≥70	−0.050	0.438
<70	0.024	0.568
Chemotherapeutic regimens		
Cisplatin-containing regimen	−0.064	0.270
Carboplatin-containing regimen	−0.087	0.390
Non-platinum regimen*	0.146	0.033
CAV-based regimen	−0.038	0.570

All analyses were weighted by sample size.

TRT, thoracic radiotherapy; CAV, cyclophosphamide, doxorubicin and vincristine.

* Regression coefficient means a slope of the fitted line in each subgroup.

** The median score was used as a cutoff level for each subclassification.

**Figure 5:**
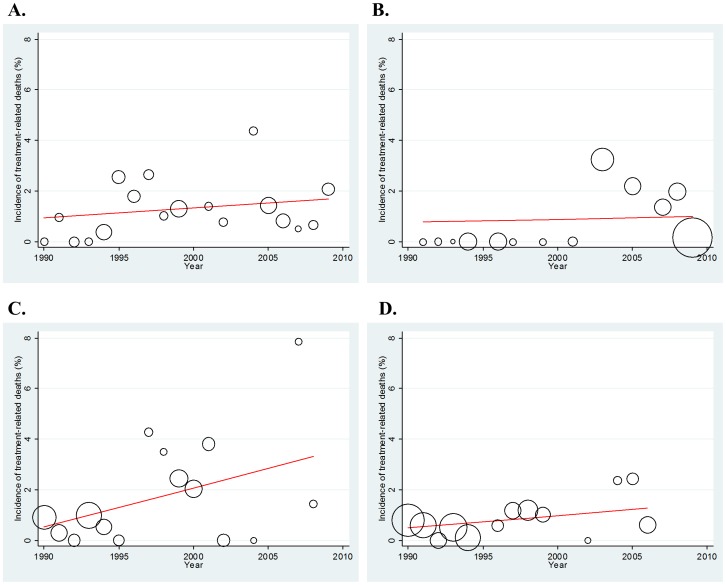
Time trend in the incidence of TRDs in relation to FN (febrile neutropenia). All analyses were weighted by sample size. A. Cisplatin-containing regimen. B. Carboplatin-containing regimen. C. Non-platinum-regimen. D. CAV (cyclophosphamide, doxorubicin and vincristine)-based regimen.

Because FN was the most common cause of fatal toxicity during chemotherapy, we focused specifically on the incidence and pattern of FN-related deaths. Overall, there was no significant time trend in TRD, with a regression coefficient of 0.035 and p-value of 0.259 (Fig. 4B). Through the entire period, the proportion of FN-related deaths was similar across the four regimens (cisplatin-based 0.649%, carboplatin-based 0.652%, non-platinum 0.645%, and CAV-based regimens 0.704%). However, the pattern of the time trend was different among the regimens (Fig. 5A D). Non-platinum regimens were associated with a significant increase in death over the years, with a 0.155% increase per year (regression coefficient = 0.155; p = 0.037; Fig. 5C), while no yearly change in the proportion was observed for the other treatment regimens (cisplatin-, carboplatin- and CAV-based regimens; p = 0.337 [Fig. 5A], 0.857 [Fig. 5B], and 0.123 [Fig. 5D], respectively).

## Discussion

We found that the incidence of overall TRDs tended to decrease over the past two decades, although it was not statistically significant (p = 0.15; Fig. 4A). In contrast, the incidence of FN-related death was fairly stable (Fig. 4B). Additionally, stratified by treatment regimen, non-platinum chemotherapy produced an increased incidence of both TRD (Table 2) and FN-related deaths (Fig. 5C) year by year.

In this study, the overall TRD incidence seems to have decreased over the last two decades, with a regression coefficient of −0.138, meaning a decrease of 0.138% per year and 2.76% per two decades (Fig. 4A). This phenomenon might be partly correlated with the observation that the number of trials designed to assess TRT, which included potentially induced fatal pulmonary fibrosis, decreased over the years, with a regression coefficient of −0.162 (0.162% decrease per year; p = 0.042). Another hypothesis is the improvement in supportive care. In NSCLC, even in patients allocated to the best supportive care alone arm, the median survival time was prolonged [14]. Similarly, in SCLC, supportive care improved over time, resulting in a decrease in the incidence of overall TRD. Further exploration is warranted to clarify the essential factors that contributed to this trend.

On the other hand, FN-related death was similar over the study period (Fig. 4B). One possible reason for this is that chemotherapeutic agents with relatively high myelotoxicity, such as etoposide or anthracyclines, have been repeatedly studied in clinical trials over the past two decades in SCLC [3], [15], [16]. Second, one would wonder consider the potential impact of the use of granulocyte-colony stimulating factor (G-CSF) on reduction in the risk of FN-related death [17], but G-CSF has been used in phase III trials since the early 1990s, corresponding approximately to the beginning of the target period investigated here [18]–[20]. Thus, G-CSF usage would likely have equally influenced the incidence of FN-related deaths throughout the study period. Further, controversy persists as to the impact of the prophylactic or routine use of G-CSF on clinical outcome, including treatment-related and overall mortality [21], [22]; there is as yet no definitive evidence regarding the impact of its use on outcome. Finally, we have no definitive data to validate the above hypotheses. Novel agents that possess less myelotoxic profiles should be developed to decrease FN-related deaths.

Meanwhile, both the overall incidence of TRD and incidence of FN-related death have increased in non-platinum regimens over the years (Table 2 and Fig. 5C). Most of the non-platinum regimens investigated here consisted of multiple agents (i.e., alternating regimens, switching regimens, and combination regimens with three or more drugs) [23]–[28], which seemed to be more toxic [7]. Assuming that the proportion of FN-related deaths accounted for a large fraction of overall TRDs, the overall TRD incidence in non-platinum regimens may have simply increased in accordance with the increase in FN-related deaths. The absolute number of trials investigating non-platinum regimens has decreased; thus, these findings seem to have less importance for clinical practice.

Our study has several limitations. First, this analysis tried to cast a wide net to capture several heterogeneous studies for the database, and the results of this study have several potential confounders and a degree of uncertainty. Second, our analyses were not based on individual patient data. Differences in patient clinical characteristics, unlike differences in the characteristics of the trial arms (chemotherapy regimen), would directly have affected the toxicity profiles. Third, a publication bias may exist. Severely toxic agents or regimens may not have been reported, resulting in an underestimation of the TRD incidence. To reduce this bias, we included both published and unpublished (abstract only) trials. Fourth, actual TRD numbers in this study seemed to be low compared with that was seen in clinical practice [29]. One explanation for this discrepancy may be that the patients eligible in such clinical studies generally tend to have better general conditions than those patients treated in clinical practice. Another explanation is that, in clinical trials, investigators might tend to produce “positive” results; that is, they would deal with true treatment-‘related’ deaths as treatment-‘unrelated’ deaths unconsciously. Thus, observed TRD numbers in the clinical trials might be smaller than the true value.

Finally, the definition of TRD and/or FN-related death might have been somewhat vague. We initially defined both TRD and FN-related death in this study as described in Methods section. However, all the trials we included here did not have identical TRD and FN definitions, which was the major limitation in our abstracted data-based analysis. Given that mentioned above, all our results should be interpreted cautiously.

In conclusion, the overall TRD proportion was low and has decreased quite gradually, but is still not negligible in phase III trials for SCLC. Physicians should be aware of these trends and do their best to reduce the risk of fatal toxicity.

## Supporting Information

File S1 **The list of 97 trials included in this study and its characteristics.**
(DOCX)
