# Supplementary material for: Treatment-Related Death in Patients with Small-Cell Lung Cancer in Phase III Trials over the Last Two Decades
Source: PLoS One. 2012 Aug 6;7(8):e42798. doi: 10.1371/journal.pone.0042798 (PMC3412813; doi:10.1371/journal.pone.0042798)
Supplement: File S1 — The list of 97 trials included in this study and its characteristics. (DOCX) [file pone.0042798.s001.docx]

| Author | Published year | Year of study initiation | No. of enrolled pts | No. of randomized pts |
| --- | --- | --- | --- | --- |
| Socinski MA | 2009 | 2006 | 908 | 908 |
| Lee SM | 2009 | 2003 | 724 | 716 |
| Lara PN Jr | 2009 | 2002 | 671 | 652 |
| Heigener DF | 2009 | 2000 | 79 | 79 |
| Dimitroulis J | 2008 | 2003 | 108 | 108 |
| Lee SM, | 2009 | 1999 | 241 | 241 |
| Hermes A | 2008 | 2001 | 220 | 208 |
| Baka S | 2008 | 1999 | 280 | 280 |
| Sculier JP | 2008 | 1993 | 214 | 204 |
| Leyvraz S | 2008 | 1997 | 145 | 145 |
| de Jong WK | 2007 | 1999 | 203 | 201 |
| Pujol JL | 2007 | 2000 | 119 | 92 |
| Okamoto H | 2007 | 1998 | 220 | 208 |
| Buchholz E | 2007 | 1996 | 86 | 86 |
| Spiro SG | 2006 | NR | 325 | 325 |
| Eckardt JR | 2006 | 2001 | 784 | 784 |
| Grote T | 2005 | 1993 | 224 | 224 |
| Ignatiadis M | 2005 | 2000 | 284 | 284 |
| Cao KJ | 2005 | 1990 | 51 | 45 |
| Niell HB | 2005 | 1998 | 587 | 565 |
| Lorigan P | 2005 | 1994 | 318 | 318 |
| Blackstock AW | 2005 | 1987 | 114 | 114 |
| McClay EF | 2005 | 1993 | 319 | 307 |
| Artal-Cortes A | 2004 | 1994 | 411 | 411 |
| Altinbas M | 2004 | 1998 | 84 | 84 |
| Reck M | 2003 | 1998 | 614 | 608 |
| Ardizzoni A | 2002 | 1994 | 244 | 244 |
| Ettinger DS | 2002 | 1988 | 139 | 135 |
| Takada M | 2002 | 1991 | 231 | 228 |
| Noda K | 2002 | 1995 | 154 | 154 |
| Tjan-Heijnen VC | 2001 | 1900 | 163 | 161 |
| Sculier JP | 2001 | 1993 | 243 | 233 |
| Mavroudis D | 2001 | 1997 | 133 | 133 |
| Pujol JL | 2001 | 1996 | 226 | 226 |
| Johnson PW | 2001 | 1996 | 84 | 84 |
| Gatzemeier U | 2000 | NR | 280 | 280 |
| Thatcher N | 2000 | 1993 | 403 | 403 |
| Urban T | 1999 | 1988 | 460 | 457 |
| Jeremic B | 1999 | 1988 | 210 | 210 |
| Bonner JA | 1999 | NR | 324 | 311 |
| Murray N | 1999 | 1992 | 220 | 219 |
| Lebeau B | 1999 | 1988 | 164 | 156 |
| Urban T | 1999 | 1985 | 434 | 394 |
| Ruotsalainen TM | 1999 | 1990 | 219 | 219 |
| Turrisi AT 3rd | 1999 | 1989 | 419 | 417 |
| Ueoka H | 1998 | 1988 | 143 | 143 |
| Furuse K | 1998 | 1991 | 228 | 227 |
| Wood L | 1998 | 1992 | 135 | 130 |
| Tummarello D | 1997 | 1990 | 140 | 140 |
| Work E | 1997 | 1981 | 199 | 199 |
| Pujol JL | 1997 | 1991 | 125 | 125 |
| Jeremic B | 1997 | 1905 | 170 | 107 |
| Fukuoka M | 1997 | 1989 | 63 | 63 |
| Zarogoulidis K | 1996 | 1991 | 90 | 90 |
| Postmus PE, | 1996 | 1988 | 148 | 148 |
| James LE | 1996 | 1998 | 167 | 167 |
| Lassen U | 1996 | 1985 | 484 | 484 |
|  | 1996 | 1989 | 310 | 310 |
| Rowland KM Jr | 1996 | 1990 | 252 | 243 |
| Miller AA | 1995 | 1990 | 319 | 306 |
| Bunn PA Jr | 1995 | 1989 | 230 | 215 |
| Gregor A | 1995 | 1988 | 37 | 37 |
| Woll PJ | 1995 | 1990 | 70 | 65 |
| Sculier JP | 1995 | 1990 | 243 | 243 |
| Joss RA | 1995 | 1984 | 415 | 415 |
| Hamm J | 1994 | NR | 240 | 235 |
| Ihde DC | 1994 | 1983 | 90 | 90 |
| Souhami RL | 1994 | 1988 | 438 | 438 |
| Skarlos DV | 1994 | 1987 | 147 | 147 |
| Clark PI | 1994 | 1986 | 94 | 94 |
| Gatzemeier U | 1994 | NR | 374 | 374 |
| Kosmidis PA | 1994 | NR | 147 | 147 |
| Maksymiuk AW | 1994 | 1987 | 571 | 552 |
| Veronesi A | 1994 | 1986 | 139 | 136 |
| Bleehen NM | 1993 | 1985 | 491 | 491 |
| Van Hoef ME | 1993 | 1989 | 26 | 26 |
| Milroy R | 1993 | NR | 226 | 220 |
| Johnson DH | 1993 | 1982 | 386 | 369 |
| Lebeau B | 1993 | 1983 | 320 | 303 |
| Erkisi M | 1993 | 1987 | 113 | 113 |
| Murray N | 1993 | 1985 | 332 | 308 |
| Monnet I | 1992 | 1988 | 60 | 60 |
| Roth BJ | 1992 | 1985 | 477 | 437 |
| Miyamoto H | 1992 | 1986 | 92 | 92 |
| Mattson K | 1992 | 1982 | 410 | 237 |
| Wampler GL | 1991 | 1983 | 170 | 170 |
| Fukuoka M | 1991 | 1985 | 300 | 288 |
| Miyamoto H | 1991 | 1985 | 92 | 89 |
| Wolf M | 1991 | 1985 | 334 | 321 |
| Smith AP | 1991 | NR | 95 | 95 |
| Jones AL | 1991 | 1987 | 104 | 104 |
| Kraft A | 1990 | 1983 | 415 | 97 |
| Ettinger DS | 1990 | 1982 | 628 | 577 |
| Nikkanen V | 1990 | 1983 | 80 | 80 |
| Goodman GE | 1990 | 1982 | 400 | 388 |
| Jett JR | 1990 | 1979 | 243 | 231 |
| Sculier JP | 1990 | NR | 221 | 201 |

Abbreviation; NR, not recorded.
